# Purification of replicating pancreatic β-cells for gene expression studies

**DOI:** 10.1038/s41598-017-17776-2

**Published:** 2017-12-13

**Authors:** Reyes Carballar, Maria de Lluc Canyelles, Claudia Fernández, Yasmina Martí, Sarah Bonnin, Esther Castaño, Eduard Montanya, Noèlia Téllez

**Affiliations:** 1 CIBER of Diabetes and metabolic diseases, CIBERDEM, Madrid, Spain; 2 Bellvitge Biomedical Research Institute, IDIBELL, Barcelona, Spain; 3 University of Barcelona, Barcelona, Spain; 4 Centre for Genomic Regulation (CRG), The Barcelona Institute of Science and Technology and UPF, Dr. Aiguader 88, Barcelona, 08003 Spain; 5 Centres Científics i Tecnològics de la UB, Barcelona, Spain; 6 Endocrine Unit, Hospital Universitari de Bellvitge, Barcelona, Spain

## Abstract

β-cell proliferation is a rare event in adult pancreatic islets. To study the replication-related β-cell biology we designed a replicating β-cells sorting system for gene expression experiments. Replicating β-cells were identified by EdU incorporation and purified by flow cytometry. For β-cell separation islet cells were sorted by size, granularity and Newport Green fluorescence emission that was combined with emitted fluorescence for EdU-labelled replicating cells sorting. The purity of the resulting sorted populations was evaluated by insulin staining and EdU for β-cell identification and for replicating cells, respectively. Total RNA was isolated from purified cell-sorted populations for gene expression analysis. Cell sorting of dispersed islet cells resulted in 96.2% purity for insulin positivity in the collected β-cell fraction and 100% efficiency of the EdU-based cell separation. RNA integrity was similar between FACS-sorted replicating and quiescent β-cells. Global transcriptome analysis of replicating *vs* quiescent β-cells showed the expected enrichment of categories related to cell division and DNA replication. Indeed, key genes in the spindle check-point were the most upregulated genes in replicating β-cells. This work provides a method that allows for the isolation of replicating β-cells, a very scarce population in adult pancreatic islets.

## Introduction

Pancreatic β-cells are the unique cell type in the body that produces and secretes insulin in response to small variations in blood glucose levels to tightly control systemic glucose homeostasis. Β-cell mass reduction is a central event in the development of type 1 and type 2 diabetes, and β-cell regeneration is a potential curative treatment of the disease.

Β-cell replication is the primary mechanism of β-cell mass expansion in adult individuals, and represents a target for diabetes treatment to increase the functional β-cell mass. However, β-cell proliferation is a rare event in adult pancreatic islets^1–5^ and varies within a range of 0.4% to 6% of β-cells per day depending on age^5^. This low proportion of replicating β-cells inside the islet precludes molecular analysis of β-cell replication-related pathways in entire islets, since it may be masked by the highly abundant post-replicative islet cells. Recently, Klochendler and colleagues have developed a transgenic mouse model where cycling cells throughout the body become GFP-labelled allowing cell sorting of live replicating cells^6^. Despite the advantages provided by this system, its use is restricted to the study of β-cell replication in mouse islets.

On the other hand, nucleoside analogues have been extensively used for the identification of replicating cells. They are incorporated into the replicating DNA strands during the S phase of the cell cycle, and the most extensively used are BrdU, CldU and IdU. In order to visualize the incorporated nucleosides, all of them require the use of DNA denaturation facilitating sterical access of antibodies to the nucleosides. Alternatively, 5-ethynyl-20-deoxyuridine (EdU) is structurally similar to the natural nucleoside in which a terminal alkyne group replaces the methyl group in the 5th position. EdU detection is based on a copper-catalyzed covalent reaction between a dye-conjugated azide and the alkyne group of the EdU, known as Click chemistry^7^. The small sized dye-azide complex allows for efficient EdU detection avoiding harsh conditions that degrade the structure of the cells^8^.

Here, we sought to develop a method for replicating β-cells sorting based on EdU incorporation, suitable for global gene expression analysis and applicable to most of experimental designs and animal species.

## Methods

### Islet isolation and culture

FELASA guidelines and recommendations for the use of laboratory animals were followed (European and local government guidelines) and animal procedures were reviewed and approved by the Animal Research Committee of the University of Barcelona (identification number: DAAM 7082).

Islets from young adult male Wistar rats (Janvier, Saint Berthevin, France) were isolated by collagenase (Collagenase P; Boehringer Mannheim Biochemicals, Mannheim, Germany) digestion of the pancreas as previously described^9^. Isolated islets were hand-picked under a stereomicroscope two or three times, until a population of pure islets was obtained. Islets were cultured in RPMI 1640 (Sigma Immunochemicals, St Louis, MO, USA) supplemented with 100 U/ml penicillin and 100 mg/ml streptomycin containing 10% heat-inactivated foetal bovine serum (FBS) at 37 °C in a humidified atmosphere with 5% CO2. Thymidine analogues, 5-Bromo-2′-deoxyuridine (BrdU, Sigma) and 5-Ethynyl-2′-deoxyuridine (EdU, Molecular probes, Life Technologies, Eugene, OR, USA) were added into the culture medium at final concentration of 10 µM. For the analysis of continuous labelling with EdU, islets were cultured in 5.5 mM or 22.2 mM glucose with or without EdU for 7 and 14 days. Culture medium was replaced on a daily basis and BrdU was added to the islets for the last 24 h of culture. For replicating β-cells sorting, islets were cultured in 5.5 mM glucose with EdU overnight starting on the day of isolation.

### Cell dispersion and labelling

In order to strengthen β-cell identification, dispersed cells were labelled with Newport Green DCF, diacetate (NG, Invitrogen, Carlsbad, CA, USA). The diacetate form of NG requires esterase-mediated cleavage for subsequent binding to Zn^2+^. Once cleaved, NG becomes membrane-impermeant and is retained inside live β-cells^10^. For replicating cell identification, the DNA-incorporated EdU was detected by azide alkyne Huisgen cycloaddition reaction. The EdU contains an alkyne which can be reacted with an azide-containing detection reagent, to form a stable triazole ring (Click reaction), eliminating the use of antibodies for detection and subsequent major permeabilization-based protocols^7^.

Cultured islets were washed with Ca^2+^ and Mg^2+^-free PBS prior trypsinization. Islets were disaggregated by gentle continuous pipetting in trypsin–EDTA 0.05% (Sigma) with DNase (1 mg/ml) (RQ1 RNases-Free DNase, Promega, Madison, WI, USA) for 6 min. in a water bath at 37 °C^11^. Dispersed islet cells were washed once with PBS and incubated with 25 µM NG at 37 °C for 30 min, protected from light. Following one 1% BSA-PBS wash, EdU labelling was performed by means of the Click-iT® EdU Alexa Fluor®647 Flow cytometry Assay Kit (Invitrogen, Eugene, OR, USA). Briefly, cells were fixed in 2% paraformaldehyde–PBS at room temperature (RT) for 15 min., and washed once with 1% BSA-PBS before mild permeabilization with a saponin-based reagent for additional 15 min. at RT. The click reaction buffer was prepared following the manufacturer’s instructions and added to the washed cells for 30 min. at RT. Prior to flow cytometry, labelled cells were washed twice with FACS buffer (PBS, 2 mM EDTA, 0.5%BSA) and transferred into 35 µm nylon mesh cell strainer caped-tubes (BD biosciences, Erembodegem, Belgium). 400 units of RNasin plus RNase inhibitor (Promega) were added to the buffer or solution used in each step from the cell fixation with PFA until cell sorting.

### Cell sorting

Unstained cells and FMO (fluorescence minus one), treated in the same way, were used as negative control in each experiment and served as autofluorescence control. Sorting was done using a Beckman-Coulter MoFlo Astrios cell sorter equipped with a 100-μm flow tip and operated at a sheath pressure of 25 psi. The laser illumination power was set to 150 mW for NG excitation at 488 nm, and to 100 mW for Alexa 647 excitation at 640 nm. The barrier filters were 517/26 nm for NG, and 671/30 nm for EdU Alexa 647 fluorescence. Before cell sorting, cells were filtered through a 35 μm strainer to eliminate all remaining cell clumps. Initially, doublets are excluded by using pulse processing (FSC-H vs. FSC-A) followed by the exclusion of debris gating on a two physical parameter dot plot (FSC/SSC). Β-cells were gated as FCS^hi^/SSC^hi^/NG^hi^. Mitotic and post-mitotic cell subpopulations were selected as EdU+ or EdU- respectively. An average sorting rate of 200 events per second was maintained. Sorted cells were collected in 150 μl of PKD buffer (Qiagen).

### Immunofluorescence

Cultured islets were fixed overnight in 4% paraformaldehyde-PBS at 4 °C, embedded in paraffin, sectioned and immunostained after deparaffinization and rehydration.

Β-cell replication in cultured islets was determined by BrdU incorporation (Cell proliferation kit, GE Health Care, Amersham, UK), and β-cell apoptosis by TUNEL assay (*In Situ* Cell Death Detection Kit, ApopTag®, Intergene, Oxford, UK) combined with insulin immunofluorescence (1/50, SC-9168, Santa Cruz Biotechnology, Inc., Santa Cruz, CA) as previously described^12^.

For EdU detection in cultured islets the click chemistry-based Click-iT EdU assay (Click-iT® EdU Alexa Fluor® 594 Imaging Kit; Molecular Probes, Life Technologies) was used according to the manufacturer’s instructions. Briefly, sections were permeabilized with saponin and incubated with the Click-iT reaction cocktail for 1 h at room temperature. This labelling method was combined with insulin immunofluorescence.

Β-cell purity in sorted populations was determined by insulin and c-peptide immunofluorescence as follows. Sorted populations were directly collected in 1% BSA-PBS, washed twice with PBS and incubated overnight with the rabbit anti-insulin antibody (1/100) or mouse-anti c-peptide antibody (1/50, Abcam) diluted in 1% BSA-PBS + 0.3% triton. Donkey anti-rabbit Alexafluor-488 labelled antibody (1/400) was used for β-cell visualization. Cells were loaded into the haemocytometer for counting. At least 200 cells were counted for each sorted population. The experiment was replicated 8 times with islets isolated from 8 different rats.

### RNA isolation, cDNA synthesis and qPCR

Total RNA was extracted with RNeasy FFPE kit (Qiagen, Crawley, UK) according to manufacturer’s instructions with the following variation. Sorted cells were directly collected in PKD buffer, immediately treated with 200 μg/ml proteinase K (Qiagen) at 56 °C for 3 h with continuous shaking. This modification significantly increased the RNA extraction yield. mRNA was linearly amplified with the Arcturus® RiboAmp® HS PLUS RNA Amplification Kit (Arcturus, Life Technologies, Foster City, CA, USA) or Complete Whole Transcriptome Amplification Kit (Sigma WTA2) following the manufacturer’s protocol. RNA quality was assessed by using the Agilent RNA Nano kit in the Bioanalyzer 2100 (Agilent Technologies, Inc., Palo Alto, CA).

cDNA synthesis was performed from 200 ng of amplified RNA using the High Capacity cDNA Reverse transcription kit (Applied Biosystems, Life Technologies). qPCR was run in a 7900HT Fast Real-Time PCR system (Applied Biosystems). Gene expression data was analysed with the free-access software Gene Expression Suite v1.0.3 (Applied Biosystems). A full listing of assays, gene names and assay identification numbers is given in Table 1.

**Table 1:** Gene expression assays from Applied Biosystems used for real-time qPCR.

	**Gene Symbol**	**Gene Name(s)**	**Assay ID**
Target genes	Acta2	smooth muscle alpha-actin	Rn01759928_g1
	Amy2a3	amylase 2a3	Rn00821330_g1
	Bmi1	Bmi1 polycomb ring finger oncogene	Rn01487369_g1
	Ezh2	enhancer of zeste homolog 2 (Drosophila)	Rn01500681_mH
	Foxo1	forkhead box O1	Rn01494868_m1
	Gcg	glucagon	Rn01485212_m1
	Hdac5	histone deacetylase 5	Rn01399498_g1
	Ins2	insulin 2	Rn01774648_g1
	Krt20	keratin 20, type1	Rn00597548_m1
	Mki67	antigen identified by monoclonal antibody Ki-67	Rn01451441_g1
	Pcam1	platelet/endothelial cell adhesion molecule 1	Rn01467262_m1
	Pcna	proliferating cell nuclear antigen	Rn00574296_g1
	Plk1	Polo Like Kinase 1	Rn00690926_m1
	Ppy	Pancreatic polypeptide	Rn00664680_g1
	Pttg1	pituitary tumor-transforming 1/securin	Rn00574373_m1
	Ska3	Spindle And Kinetochore Associated Complex Subunit 3	Rn01488687_m1
	Slc16a1	monocarboxylate transporter 1	Rn00562332_m1
	Spc25	NDC80 Kinetochore Complex Component	Rn01484156_m1
	Sst	somatostatin	Rn00561967_m1
	Vim	vimentin	Rn00667825_m1
Housekeeping genes	18srRNA	18S ribosomal RNA	Hs99999901_s1
	Hprt1	hypoxanthine phosphoribosyltransferase 1	Rn01527840_m1
	Rpl30	ribosomal protein L30	Rn01504620_g1
	Rps17	40S ribosomal protein S17-like	Rn00820807_g1

### Global gene expression profiling

Total RNA was amplified using the TransPlex Complete Whole Transcriptome Amplification Kit (Sigma WTA2) to generate the cDNA. Cyanine-3 (Cy3) labelled cDNA was prepared from 500 ng of double stranded cDNA using the SureTag Complete DNA Labelling kit (Agilent 5190-4240) according to manufacturer’s instructions. Dye incorporation and cDNA yield were checked with the NanoDrop ND-1000 Spectophotometer. 600 ng of Cy3 labelled cDNA was directly mixed with hybridization buffer and Agilent blocking agent incubated at 95 °C for 3 minutes and immediately transferred on ice. Then, it was hybridized to Agilent SurePrint G3 Rat Gene Expression v2 8 × 60 K Microarray (amadid ID: 074036) for 40 h at 65 °C in a rotating Agilent hybridization oven. After hybridization, microarrays were washed 1 minute at room temperature with GE Wash Buffer 1 (Agilent) and 1 minute with 37 °C GE Wash buffer 2 (Agilent), then dried immediately by brief centrifugation. Microarrays were scanned on an Agilent G2539A scanner at 3 µm resolution and 100%PMT. The intensity data of each individual hybridization were extracted and the quality was assessed with the Feature Extraction software 10.7 (Agilent). Samples were processed in triplicates: three quiescent β-cells samples, and three replicative β-cells samples. Raw data was corrected for background noise using the normexp method. Quantile normalization was applied to assure comparability between samples. Differential expression analysis was carried out on non control probes with an empirical Bayes approach on linear models. A paired test was applied, taking into account that the cells were separately sorted from 3 different islets preparations. Results were corrected for multiple testing according to the False Discovery Rate (FDR) method^13^. All pre-processing and statistical analyses were performed with the Bioconductor project in the R statistical environment, in particular the limma package^14,15^. For Gene Set Enrichment Analysis (GSEA), *rattus norvegicus* Gene Ontology gene sets were downloaded from the Gene Ontology website (www.geneontology.org/page/gene-associations/gene_association.rgd.gz) and transformed into a format suitable for the GSEA software. P-values were computed using a t-Test, using 1000 permutations (“gene_set” permutation type) and the default Signal2Noise metric. Nominal p-values were then corrected for multiple testing using the False Discovery Rate. For EnrichR analysis, probes were selected based on p-value lower than 0.001 and absolute linear fold change higher than 2. Median intensities are retrieved for a gene if it is represented by multiple probes. Those genes were fed into the EnrichR software to test for enrichment of pathways and ontologies^16^. Data have been deposited in Gene Expression Omnibus (www.ncbi.nlm.nih.gov/geo), accession number GSE104387.

### Statistical analysis

Results are expressed as means ± SEM for 4–8 independent experiments. Statistical analysis were performed using GraphPad Prism 6 software, and differences among means were evaluated using the Student’s paired t test with p < 0.05 considered significant.

### Data availability

The datasets generated during and/or analysed during the current study are available from the corresponding author on reasonable request.

## Results

### Continuous labelling with EdU *ex vivo*

**Figure 1:**
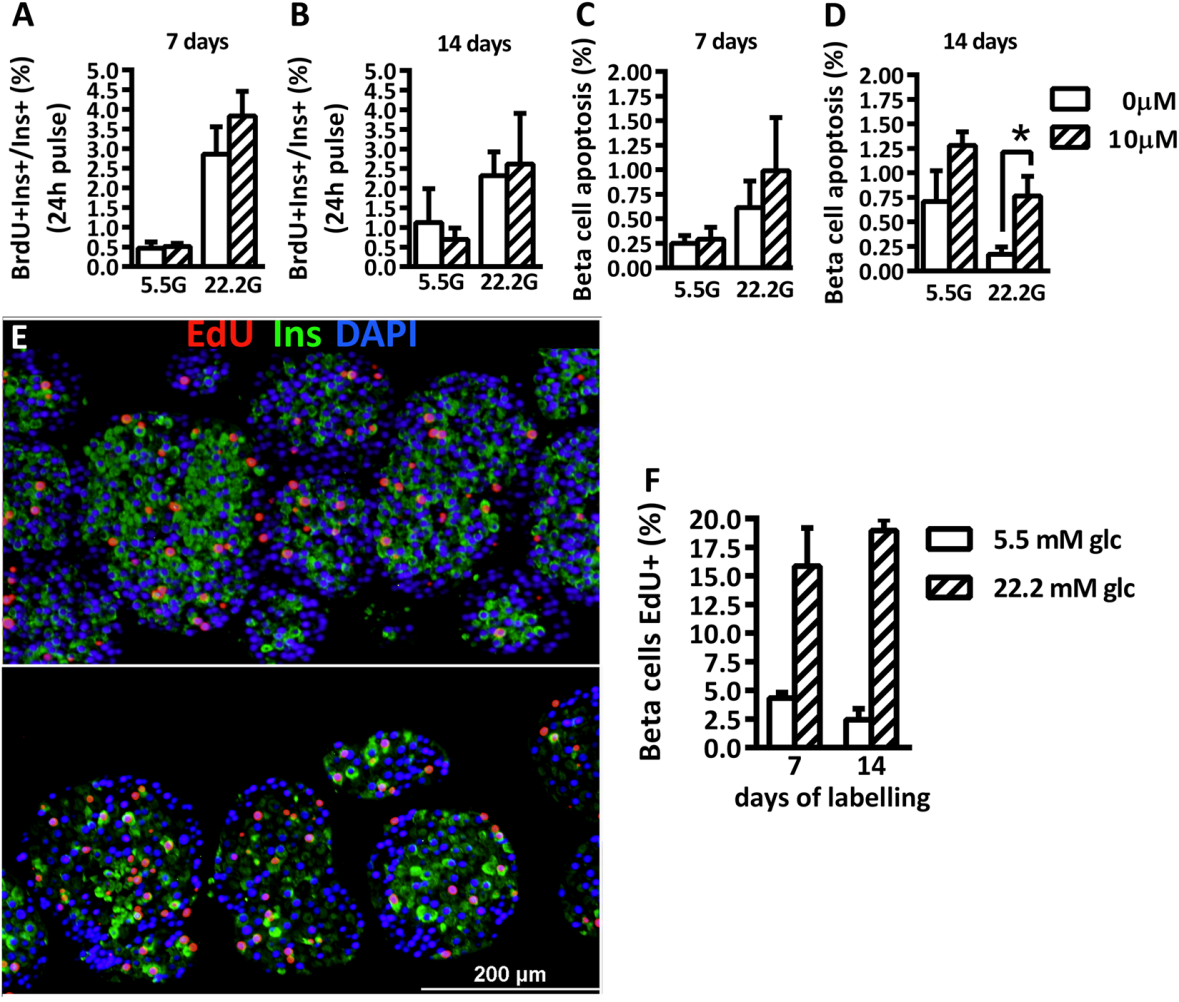
Continuous labelling of islets with EdU *ex vivo*. Rat pancreatic islets were cultured at 5.5 or 22.2 mM glucose in the presence or absence of 10 µM EdU. 10 µM BrdU was added into the culture medium for the last 24 h. (**A**,**B**) β-cell replication (BrdU incorporation) and (**C**,**D**) β-cell apoptosis (TUNEL) of islets exposed to EdU for 7 days (**A**,**C**) or 14 days (**B**,**D**). Values are means ± SEM of n = 8. *p < 0.05. (**E**) Representative image of EdU and insulin staining on islets cultured for 7 (top) or 14 days (bottom) at 22.2 mM glucose with continuous EdU labelling. (**F**) Quantification of β-cells positive for EdU.

Long term labelling of mice with halogenated thymidine analogues has been found to inhibit β-cell proliferation *in vivo* ^17^. In order to evaluate the effects of EdU on β-cell replication and viability, rat pancreatic islets were cultured with or without 10 µM EdU at basal and high glucose concentrations. B-cell replication and apoptosis were unaffected by one week exposure to EdU at both glucose concentrations (Fig. 1A,C). After 14 days exposure to EdU, β-cell replication remained unaffected but β-cell apoptosis was significantly increased (Fig. 1B,D). Accordingly, the percentage of EdU labelled β-cells at the end of the 7 days-culture was approximately 7-fold over the 1 day labelling with BrdU (5.5 mM glc and 24 h-BrdU pulse: 0.51 ± 0.08% labelling; 5.5 mM glc and 7days-EdU pulse: 4.3 ± 0.48% labelling); and longer pulse of EdU did not result in further β-cell labelling (5.5 mM glc and 14days-EdU pulse: 2.45 ± 0.94% labelling) (Fig. 1E,F). Therefore, continuous exposure of cultured pancreatic islets to EdU for one week is suitable for β-cell replication experiments, whereas longer cultures should be avoided.

### Fluorescent Activated Cell Sorting (FACS) of replicating β-cells

**Figure 2:**
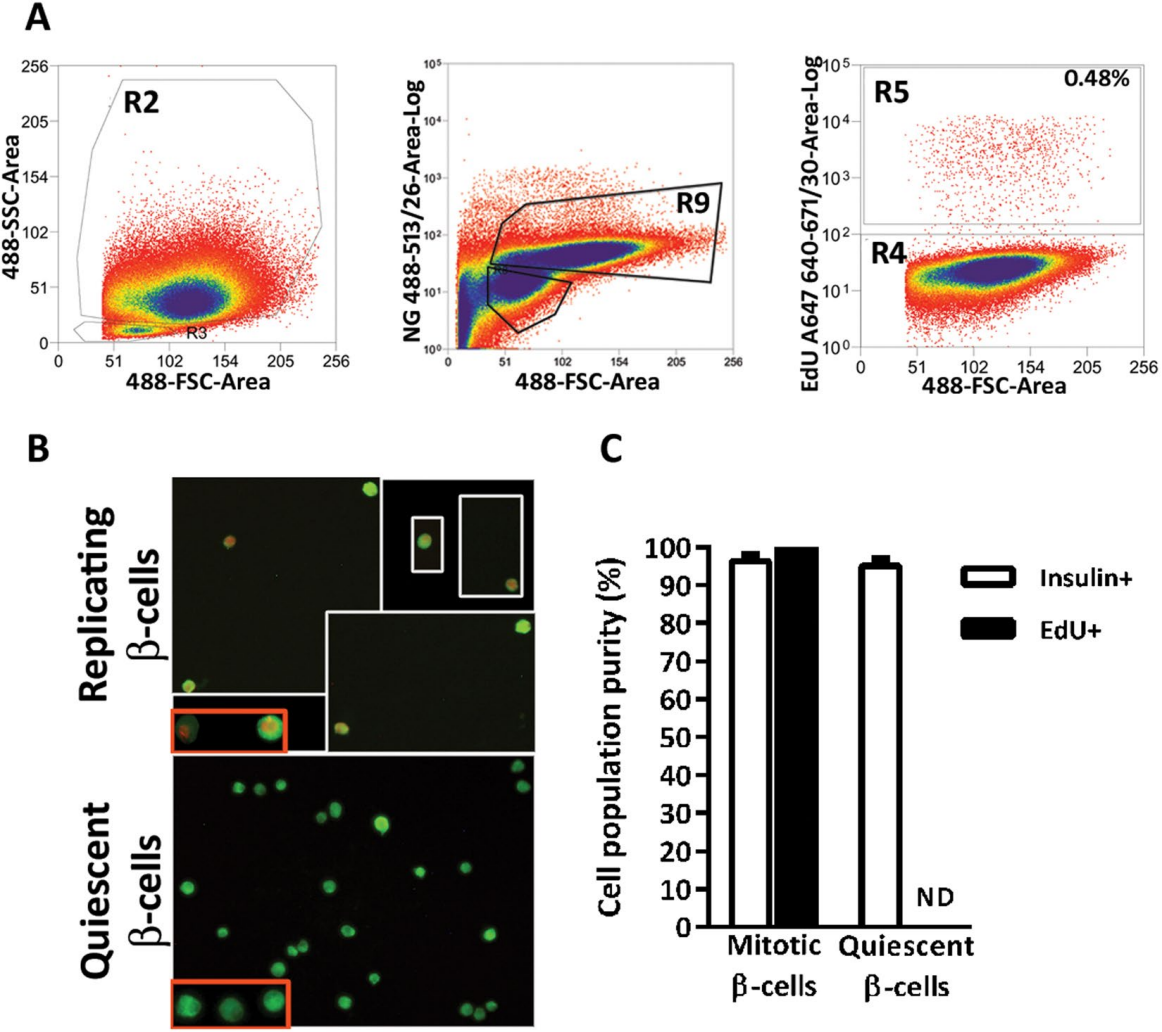
Efficiency of replicating and quiescent β-cell sorting. (**A**) Replicating and quiescent β-cells were sorted after gating on SSC-A^hi^/FSC-A^hi^ (R2) and FSC-A^hi^/NG-A^hi^ (R9) for β-cell population, and EdU647^+^ (R5) and EdU647^−^ (R4) for replicating and quiescent cells, respectively. (**B**) To assess the purity of each cell population, sorted cells were labelled with insulin, visualized and (**C**) counted. Mitotic β-cell population is illustrated as a composite of pictures from 4 different fields to show a representative number of cells. Insets framed in red show replicating (top) and quiescent (bottom) β-cells at higher magnification. ND: Non-detected. Values are means ± SEM of n ≥ 5.

Live β-cells from rat islets are efficiently purified by FACS according to size (Forward Scatter Light, FSC), granularity (Side Scatter Light, SSC) and auto-fluorescence when excited at a wavelength of 488 nm^18–21^. The use of NG, a zinc-binding fluorochrome, further improves β-cell identification^22^. EdU labelling requires cell fixation and mild permeabilization to allow the Alexa Fluor-labelled azide molecule reach the DNA-integrated thymidine analogue in replicating cells. Cell fixation and permeabilization resulted in loss of intracellular NG with subsequent poor green fluorescence in β-cells and reduced size (Supplementary Figure 1). Nevertheless, pure β-cells were obtained by sorting islet cells by size, granularity and green fluorescence emission (Fig. 2A). Four cell fractions were collected: Replicating β-cells (SSC^hi^, FSC^hi^, NG^+^, EdU^+^), Quiescent β-cells (SSC^hi^, FSC^hi^, NG^+^, EdU^−^), Replicating non-β-cells (SSC^low^, FSC^low^, NG^−^, EdU^+^) and Quiescent non-β-cells (SSC^low^, FSC^low^, NG^−^, EdU^−^). One hundred per cent of the cells collected in the replicating β-cell fraction was positive for EdU and 96 ± 1% and 98.8 ± 1.2% stained for insulin and c-peptide, respectively (Fig. 2B,C). In the non-replicating β-cell population, 100% of cells were negative for EdU and 95 ± 1%, and 99.5 ± 0.07% stained positive for insulin and c-peptide, respectively (Fig. 2B,C). In non-β-cell fractions 7.8 ± 0.1% of cells stained positive for insulin.

The number of cells collected per rat pancreas ranged from 760 to 1200 cells in the proliferative β-cell fraction and from 4 × 10^4^ to 6 × 10^4^ cells in the quiescent β-cell fraction.

### RNA isolation from quiescent and replicating sorted cells

RNA was isolated from replicating and quiescent β-cell fractions. RNA extraction from the quiescent β-cell fraction that consisted of approximately 2 × 10^4^ cells, was up to 160 ng of RNA (~8 pg RNA/cell), whereas for the replicating fraction with ≈1000 cells, the collected RNA yielded ~5 ng of RNA (~5 pg of RNA/cell). Similar results were obtained in the RNA extraction from 1000 quiescent β-cells, excluding the possibility that replicating β-cells had lower content of RNAs. Thus, the efficiency of RNA extraction correlated with the number of cells in the fraction.

**Figure 3:**
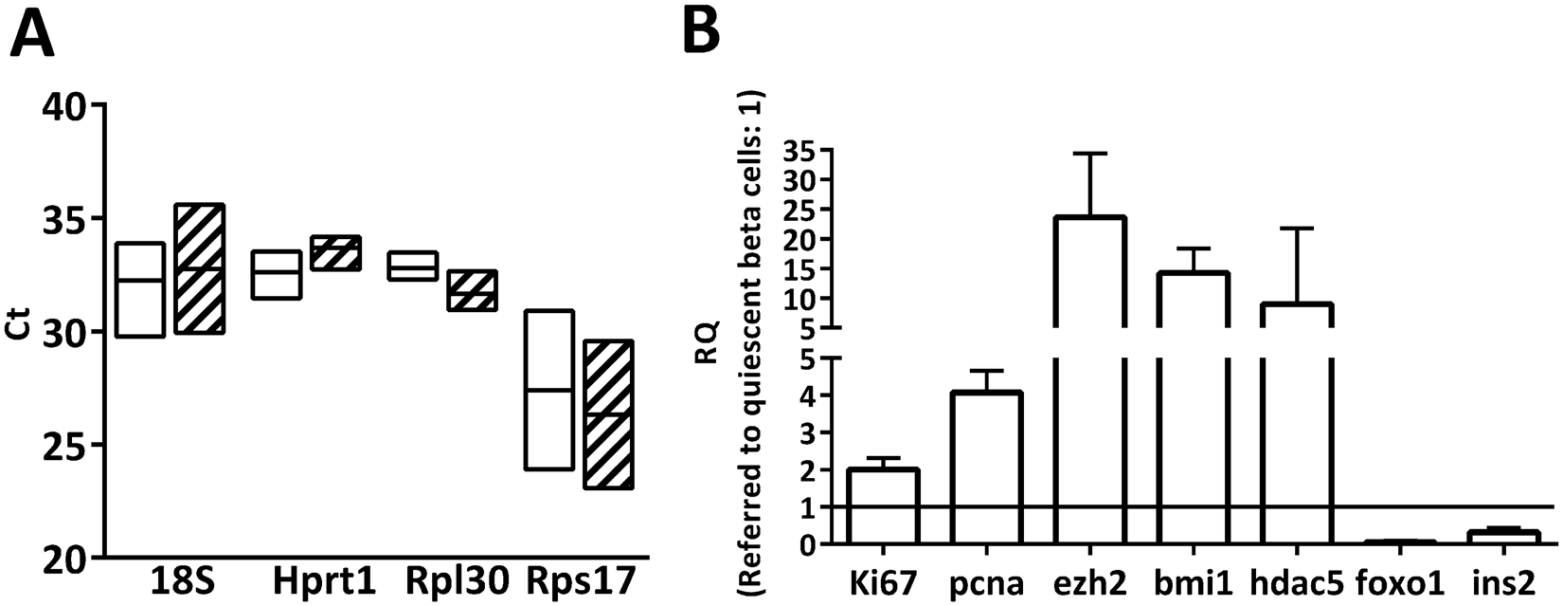
Gene expression of replicating β-cells. (**A**) Ct values of housekeeping genes used as endogenous controls amplified in replicating (empty) and quiescent β-cells (hashed). Values are represented as median [IQR] of n = 6. (**B**) Differential expression of cell-cycle related genes and insulin in replicating over quiescent β-cells. Values are means ± SEM of n = 4.

mRNA integrity analysed by the Agilent 2100 Bioanalyzer was similar between the two collected fractions, and electropherograms displayed the expected RNA mobility curve shown for other cell types^23^. For gene expression analysis, equal amounts of retro-transcribed cRNA were loaded for replicating and quiescent β-cell samples. Expression of the housekeeping genes (18*S, hpdt1, rpl30* and *rps17*) was similar between replicating and quiescent β-cells, indicating that the amount of loaded mRNA was similar in both groups (Fig. 3A).

In order to confirm the purity of the collected fractions, a panel of pancreatic cell types and proliferation markers were analysed in both fractions. Gene expression of amylase (*amy)*, keratin 20 (*krt20)* and *pecam1* were undetectable in our samples, indicating the absence of contaminating acinar, duct or endothelial cells (data not shown). However, mRNAs encoding for glucagon (gcn) and somatostatin (sst) were detected in 2 out of 11 samples from both fractions, and pancreatic polypeptide (ppy) in 6 out of 11. Finally, vimentin the major cytoskeletal component of mesenchymal cells was detectable in both β-cell fractions and levels of expression in the replicating β-cell fraction were doubled compared to the quiescent β-cell fraction. α-smooth muscle actin (acta2), a marker for the epithelial to mesenchymal transition (EMT) was undetectable.

*ki67* and *pcna* mRNAs, two established markers of cell cycle progression^24^ were significantly higher in replicating β-cells compared with the non-replicating β-cell population. Similarly, the Polycomb Repressive Complex (PRC) members, *bmi1* polycomb ring finger oncogene (*bmi1*) and the enhancer of zeste homolog 2 (*ezh2*) were upregulated in replicating β-cells (Fig. 3B). Both repressors regulate the expression of the product of the Ink4a locus (*cdkn2a)* that in turn negatively regulates cell cycle progression by targeting cdk4-cycD2^25,26^. Histone Deacetylase 5 (HDAC5), a Class IIa HDAC that is associated with cell cycle progression in several cell types^27^ was also upregulated in the replicating β-cell population. Finally, gene expression of the forkhead box protein O1 (*Foxo1*) and insulin (*ins*) was significantly downregulated in replicating β-cells. These results confirm the enrichment of replicating cells in the EdU+ β-cell fraction.

### Genes up-regulated in replicating β-cells

**Figure 4:**
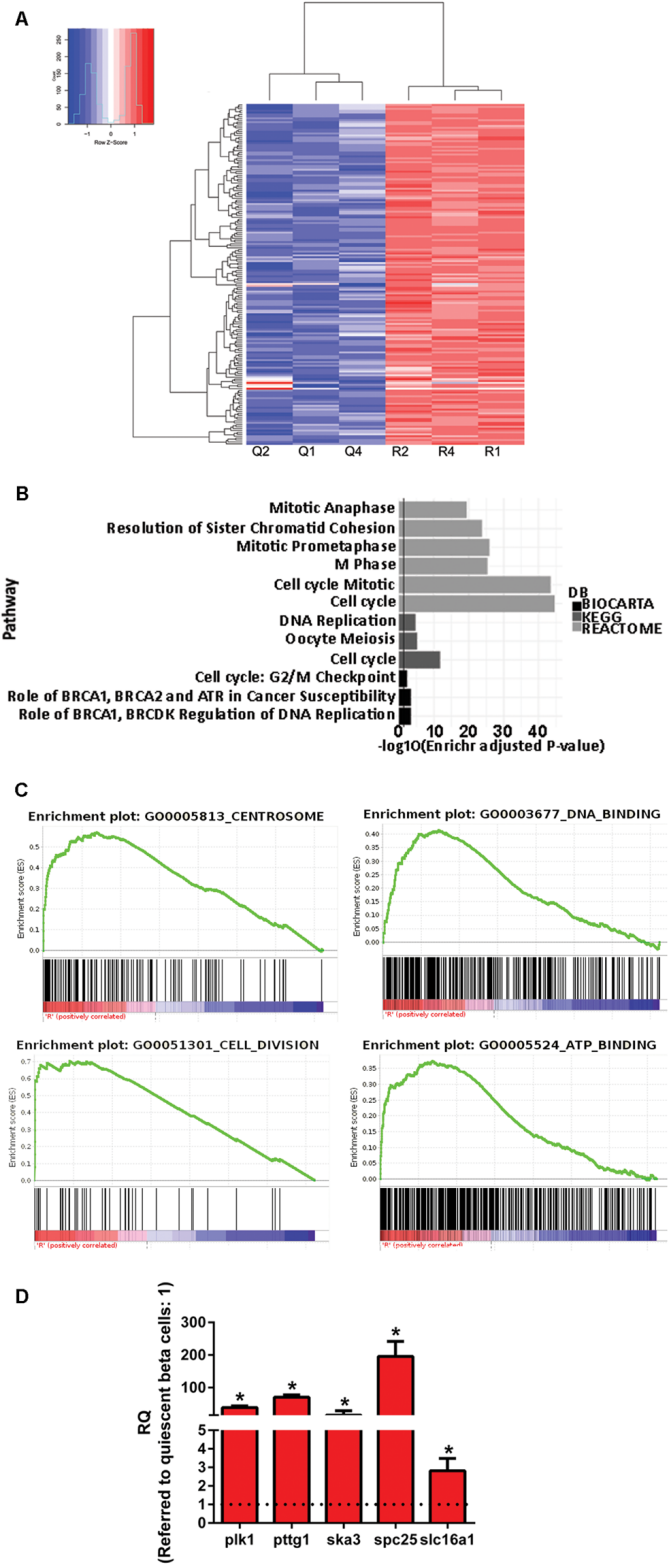
Genetic programme of replicating β-cells. (**A**) Heat-map of most changing genes. Selection according to p-value < 0.001 and absolute linear fold change > 2. Q: quiescent β-cells; R: replicating β-cells. (**B**) EnrichR analysis of most changing genes. Relevant and significantly enriched gene sets from Biocarta, KEGG and Reactome data bases are represented in the bar-plot. (**C**) Gene Set Enrichment Analysis showing selected Rat Gene Ontology data sets enriched in the replicating β-cells compared to the quiescent β-cells. (**D**) Quantitative RT-PCR determination of mRNA levels of selected genes from the top regulated transcripts unveiled in the gene array. Values are means ± SEM of n = 4. *p < 0.05 *vs* quiescent β-cells.

We next analysed the global transcriptome of replicating and quiescent β-cells using gene expression arrays. A high variation within experimental groups did not allow to select genes of interest based on the adjusted p-value. However, different selection criteria (non-corrected p-value inferior or equal to 0.05 and absolute log2 ratio superior or equal to 1) allow to extract 5500 differentially expressed genes (2043 and 3457 down- and up-regulated in the replicating β-cells, respectively). Heat-map of most changing genes revealed good clustering of the samples within each experimental group (Fig. 4A). Gene Ontology analysis of the replicating β-cell signature showed the expected enrichment of categories related to cell division and DNA replication, such as Cell Division (GO:0051301), Centrosome (GO:0005813), DNA-binding (GO:0003677) or ATP-binding (GO:0005524) (Fig. 4B,C). Comparable results were obtained with the EnrichR and GSEA software indicating the consistency of the functional analysis.

The genes most significantly upregulated in replicating β-cells are mainly involved in the control of G2-M phases of the cell-cycle (Table 2 and Fig. 4D). Interestingly, the *Slc16a1* gene that encodes for the Monocarboxilate transporter 1, which conveys pyruvate and lactate through the plasma membrane and its expression is negligible in mature β-cells, was significantly upregulated in replicating β-cells.

**Table 2:** Top 30 genes upregulated in replicating β-cells, sorted from lowest p-value.

Gene symbol	Description	FC	P value
Rrm2	Ribonucleotide reductase M2	40,52	9,72E-06
Ube2t	Ubiquitin-conjugating enzyme E2T	42,69	1,30E-05
Kif22	Kinesin family member 22	25,02	1,92E-05
Bub1	BUB1 mitotic checkpoint serine/threonine kinase	26,30	2,04E-05
Knstrn	Kinetochore-localized astrin/SPAG5 binding protein	47,61	2,38E-05
Cdca3	Cell division cycle associated 3	23,95	2,79E-05
Spag5	Sperm associated antigen 5	29,37	2,96E-05
Cdk1	Cyclin-dependent kinase 1	25,18	3,16E-05
Ckap2	Cytoskeleton associated protein 2	18,21	3,41E-05
Cep55	Centrosomal protein 55	37,72	3,78E-05
Ndc80	NDC80 kinetochore complex component	48,38	3,84E-05
Kif18b	Kinesin family member 18B	24,19	4,00E-05
Kif20b	Kinesin family member 20B	22,55	4,05E-05
Nusap1	Nucleolar and spindle associated protein 1	15,16	4,47E-05
Pttg1	Pituitary tumor-transforming 1/Securin	20,38	4,49E-05
Ncaph	non-SMC condensin I complex, subunit H, transcript variant X1	22,15	4,52E-05
Top2a	Topoisomerase (DNA) II alpha (Top2a)	15,13	4,82E-05
Tpx2	TPX2, microtubule-associated	15,63	4,85E-05
Mcm5	Minichromosome maintenance complex component 5, transcript variant X1	14,84	5,21E-05
Mastl	Microtubule associated serine/threonine kinase-like, transcript variant X1	13,74	5,38E-05
Prc1	Protein regulator of cytokinesis 1	15,36	5,43E-05
Arhgap11a	Rho GTPase activating protein 11A	15,68	6,44E-05
Ect2	Epithelial cell transforming 2	12,77	6,90E-05
Cdkn3	Cyclin-dependent kinase inhibitor 3	18,65	7,60E-05
Plk1	Polo-like kinase 1	33,58	7,85E-05
Cenpw	Centromere protein W	23,25	8,12E-05
Nek2	NIMA-related kinase 2	13,50	8,38E-05
Cenpm	Centromere protein M	22,09	8,42E-05
Bard1	BRCA1 associated RING domain 1	36,16	9,46E-05
Spc25	SPC25, NDC80 kinetochore complex component	35,49	1,03E-04

FC: Fold Change of gene expression in replicating vs quiescent β-cells.

## Discussion

This study reports a method for the purification of replicating and quiescent pancreatic β-cells for downstream gene expression analysis. This technique is based on the incorporation of EdU to the replicating DNA strands during the S phase of the cell cycle, and makes it compatible with diverse experimental designs and animal species.

Nucleotide analogues have been extensively used for the identification of replicating cells. However, long term exposure to BrdU and IdU has been associated with DNA instability, DNA stress and cell death^17^. In the present study we have determined the effects of long-term EdU exposure on β-cell replication and survival. We show that β-cell replication and viability are unaffected in cultured islets exposed to 10 μM of EdU for 7 days; whereas longer exposure has a negative impact on β-cell survival.

EdU, in contrast to other thymidine analogues such as IdU, CldU or BrdU, does not require the use of HCL-based DNA denaturation for antigen retrieval and its visualization is free from secondary antibodies^7^, which results in the use of milder and quicker protocols for its detection and preserves RNA integrity. However, cell fixation with PFA, a mandatory step in the EdU labelling by Click reaction, significantly influenced RNA extraction yield. PFA fixation is assigned to its reactivity and ability to modify proteins and nucleic acid bases resulting in RNA-protein cross-linking^28^, which compromises RNA isolation from fixed cells. Accordingly, the inclusion of a protein digestion step in the RNA extraction protocol, with the addition of Proteinase K in the cell lysis buffer, significantly improved the quality and yield of the RNA.

Islet cell sorting according to cell size, granularity and NG labelling resulted in pure β-cell preparations. Notably, amylase and keratin 20 RNAs were undetectable in β-cell preparations. However, non β-cell pancreatic islet hormones (glucagon, somatostatin and pancreatic polypeptide) RNAs were detectable in some samples, indicating that contamination with endocrine non β-cells was not completely avoided.

Differential gene expression showed the expected upregulation of cell cycle –related genes in the replicating β-cell fraction, which confirms the replicative nature of the sorted cells. Additionally, *Foxo1* was significantly downregulated in replicating β-cells. Nuclear accumulation of Foxo1 has been related to cell longevity, indeed, increased Foxo1 expression has been found in aged β-cells which are largely quiescent^29,30^. Therefore, the lower expression of *foxo1* in replicating β-cells is in accordance with the inverse relationship between Foxo1 expression and β-cell replication. Finally, insulin 2 gene expression was downregulated in replicating β-cells. In accordance with our results, recent reports have shown a significant reduction on insulin synthesis prior to β-cell replication^31,32^.

Global transcriptome of replicating and quiescent β-cell fractions showed an enrichment in cell-cycle-related categories such as centrosome, DNA-replication and DNA-binding indicating that the EdU-based sorting method efficiently provided pure preparations of replicating β-cells. Key components of the G2 and M phases of the cell-cycle were identified in replicating β-cells. Of note, *FoxM1*, *plk1* and *cenp* were upregulated in replicating β-cells. This axis has been shown to play a crucial role in insulin-stimulated β-cell proliferation^33–35^. Other regulators of the M phase of the cell-cycle such as *pttg1* (Pituitary Tumor-Transforming 1, securin), *ska3* (Spindle and Kinetochore Associated Complex Subunit 3) and *spc25* (SPC25, NDC80 Kinetochore Complex Component) with unclear roles in β-cell expansion, were also upregulated in replicating β-cells. Another gene that was significantly induced in replicating β-cells was *Slc16a1* that encodes for monocarboxilate transporter 1 (MCT1). Expression of MCT1 is specifically repressed in adult β-cells which prevents lactate and pyruvate permeability through the plasma membrane, and allows for accurate glucose-induced insulin secretion^36,37^. Thus, replicating β-cells could incur in inappropriate insulin release in response to glucose levels. Further work will reveal whether the genetic program of cycling β-cells is completely restored in daughter cells after mitosis.

In summary, we present a method that allows for the isolation of replicating β-cells, even in situations where they are very scarce. We anticipate that it will be particularly useful for the study of β-cell replication of human islets, in which replicating β-cells are infrequent.

Therefore, we expect this method to have a broad applicability in the study of β-cell proliferation in the context of diabetes and β-cell regeneration.

## Electronic supplementary material

Supplementary figure 1
