# Publisher Correction: Purification of replicating pancreatic β-cells for gene expression studies

**DOI:** 10.1038/s41598-018-24857-3

**Published:** 2018-04-25

**Authors:** Reyes Carballar, Maria de Lluc Canyelles, Claudia Fernández, Yasmina Martí, Sarah Bonnin, Esther Castaño, Eduard Montanya, Noèlia Téllez

**Affiliations:** 1 CIBER of Diabetes and metabolic diseases, CIBERDEM, Madrid, Spain; 2 Bellvitge Biomedical Research Institute, IDIBELL, Barcelona, Spain; 3 University of Barcelona, Barcelona, Spain; 4 Centre for Genomic Regulation (CRG), The Barcelona Institute of Science and Technology and UPF, Dr. Aiguader 88, Barcelona, 08003 Spain; 5 Centres Científics i Tecnològics de la UB, Barcelona, Spain; 6 Endocrine Unit, Hospital Universitari de Bellvitge, Barcelona, Spain

Correction to: *Scientific Reports* 10.1038/s41598-017-17776-2, published online 13 December 2017

This Article contains typographical errors in the Acknowledgements section.

“This work was supported by grants from the Catalan Diabetes Association (NT), University of Barcelona (NT), Carlos III Health Institute (ISCIII) PI17/00108 co-funded by FEDER funds/European Regional Development Fund (ERDF)-“a Way to Build Europe” (EM) and by CIBERDEM which is a project of ISCIII. The authors thank the CRG Genomics and Bioinformatics facilities.”

should read:

“This work was supported by grants from the Catalan Diabetes Association (NT), University of Barcelona (NT), Carlos III Health Institute (ISCIII) PI16/00462 co-funded by FEDER funds/European Regional Development Fund (ERDF)-“a Way to Build Europe” (EM) and by CIBERDEM which is a project of ISCIII. The authors thank the CRG Genomics and Bioinformatics facilities.”

