# Supplementary material for: Purification of replicating pancreatic β-cells for gene expression studies
Source: Sci Rep. 2017 Dec 13;7:17515. doi: 10.1038/s41598-017-17776-2 (PMC5727529; doi:10.1038/s41598-017-17776-2)
Supplement: Supplementary file 1 — Supplementary figure 1 [file 41598_2017_17776_MOESM1_ESM.pdf]

TITLE: Purification of replicating pancreatic β-cells for gene expression studies.

AUTHORS: Reyes Carballar[3*], Maria de Lluc Canyelles[3*], Claudia Fernández[1,2,3*], Yasmina Martí[3], Sarah Bonnin[4], Esther Castaño[5], Eduard Montanya[1,2,3,6], Noèlia Téllez[1,2,3]

Supplementary figures

Supplementary figure 1

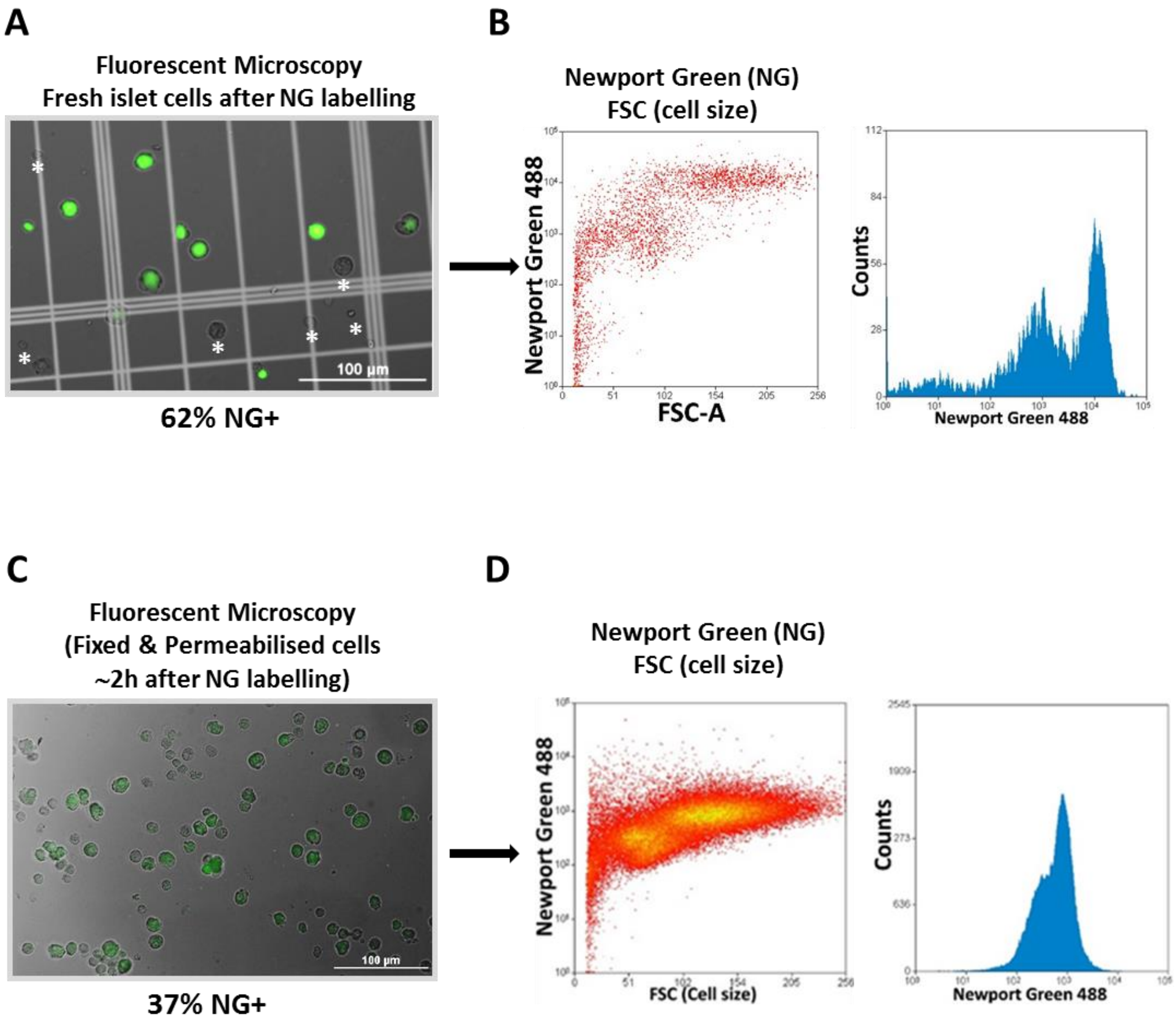

Supplementary figure 1. **Effects of PFA fixation on NPG labelling**. A-C) Fluorescent microscopy and B-D) flow cytometry-based analysis of dispersed islet cells labelled with NPG. A) Representative image and B) representative plots of NPG detection in islet cells analyzed immediately after NPG labelling. C) and D) representative image and plots of NPG detection in cells analyzed after fixation and permeabilization following NPG labelling.
